# The Implementation of Bring Your Own Device (BYOD) in Primary [Elementary] Schools

**DOI:** 10.3389/fpsyg.2016.01739

**Published:** 2016-11-15

**Authors:** Karen J. McLean

**Affiliations:** Faculty of Education and Arts, Australian Catholic UniversityBallarat, VIC, Australia

**Keywords:** BYOD, bring your own device, mobile technologies, BYOT

The increasing emphasis on one-to-one technology programs has led to schools exploring options for technology provision (Stavert, 2013). This is because of costs involved in ensuring one-to-one access to technology for all children (Cardoza and Tunks, 2014). Of interest in the current educational climate are *bring your own device* (BYOD) approaches to provision where students bring their own technology devices to school for learning. This paper considers issues around the application of BYOD approaches in primary [elementary] schools.

## Educational context

The introduction of mobile devices in schools has been met with approval from the education establishment. This is mainly due to the reported potential of these devices for supporting contemporary views of teaching and learning (Traxler, 2009). As examples of mobile devices, mobile phones and mobile tablet technologies have potential to support collaborative learning in conventional and online learning environments (Falloon, 2015). The instant access to, and flexibility of mobile devices are seen as enablers for collaborative learning (Murray and Olcese, 2011). It is these features of mobile technologies that have influenced a change in the way that technology use is viewed in primary education. Although most educators would agree that mobile technologies have the potential to transform teaching and learning practices in schools (Zurita and Nussbaum, 2004; Traxler, 2009; Hedberg, 2014) models to support this provision continue to be debated.

## Models of technology provision

Models for provision of technology have changed (see for example Alberta Education, 2012; Stavert, 2013) since computers were first introduced in schools. These changes can be seen through a shift from computer labs to learning pods, learning pods to notebooks programs, and notebook programs to one-to-one mobile technology programs. These shifts are largely attributed to sociocultural theoretical influences on technology provision. These influences were initially realized through shared learning and learning pod arrangements and are now evident in models for one-to-one access and collaborative use of technologies (Kearney et al., 2015).

Two main forms of provision for one-to-one ratios of student access have emerged. The first involves schools purchasing mobile devices, which remain on site as class sets. Limitations of this model include purchase costs for schools, information technology (IT) infrastructure required to maintain the devices and keeping track of student work throughout schooling (Nelson, 2012). The second form of provision involves student ownership of, and responsibility for these devices. This model requires parents to purchase self-sourced devices recommended by the school or through leasing arrangements instigated through school processes (Johnson, 2012; Bruder, 2014). This is referred to as the *Bring Your Own Device* (BYOD) or *Bring Your Own Technology* (BYOT) model. Using the BYOD model parents provide the technologies for their children's use in similar ways to other educational resources such as books (Falloon, 2015).

## Arguments for the implementation of BYOD in primary schools

The BYOD model is reported as benefiting schools through relieving the cost pressure for one-to-one technology provision (Cardoza and Tunks, 2014) and providing relief for technology support (Nelson, 2012). Several adaptions of this model have emerged to support parents in the process of purchasing mobile devices for educational use. These include parents having responsibility for (a) purchase, maintenance, and software installation; (b) purchase, but the device is managed by the school; and (c) purchase, but varying levels of maintenance and software installation are supported by the school (Sweeney, 2012).

The ubiquity of mobile devices and pervasive ownership from all socioeconomic groups provide compelling reasons for the adoption of BYOD in schools (Johnson, 2012; Stavert, 2013). Although BYOD in primary schools may draw on these elements of students' lives and provide continuity across school and home learning contexts (Lai et al., 2013) the impact of BYOD on these contexts is less certain.

Reported benefits associated with BYOD in schools include high levels of student engagement through interactive assignments, the use of a range of apps to teach core curriculum skills and independent inquiry learning opportunities (Bruder, 2014). This engagement is attributed to student-centered pedagogical approaches that have emerged in response to the non-standardized learning environments that are created when students bring their own devices to school for learning (Sweeney, 2012). Other benefits of BYOD practices in schools are reported by Song (2014). In this Hong Kong study students' perceptions of learning through participation in a BYOD science inquiry program were investigated. Although this study was limited to year 6 students in one school, the findings support claims that BYOD practices contribute to student engagement and support learning through student-centered inquiry approaches.

BYOD in schools is described as contributing to flexible and collaborative learning environments (Johnson et al., 2014). For example, Clark (2013) describes the benefits for students in US county schools of engaging in BYOD practices in terms of creativity, critical thinking, communication, collaboration, confidence, citizenship, and community. Clark (2013) argues that the implementation of BYOT practices contributed to transforming the traditional classroom through empowering teachers and students using personalized learning approaches (Clark, 2013). Similar innovative practices are described by Falloon (2015) in New Zealand research where benefits of using iPads extended into the home. Findings such as these lend support to arguments for BYOD in schools but also suggest a need to examine the broader influences of BYOD on family and school practices.

## Arguments against BYOD in primary schools

Constraining factors influencing the implementation of BYOD in primary schools include the legal obligations of schools around the support and provision of these devices for all students (Bathon, 2013). Approaches to ensuring security and appropriate use of devices outside of school (Fogarty and Carr, 2014) include the use of guidelines to improve network management (Sweeney, 2012) and the use of filters and controls (Ullman, 2011). Despite this, the extent to which schools can control security and out of school use is unclear.

A further argument against the implementation of BYOD in primary schools' centers on equitable access to mobile devices for all children (Stager, 2011; Johnson, 2012). For example, variations in models purchased, applications installed on individual devices and subscriptions to applications with controlled access to levels. One way of addressing this issue is through a combination of BYOD and school-based models of mobile technology provision. Using these approaches schools purchase additional mobile technologies to supplement one-to-one ownership in efforts to ensure that all children have access to a device for learning (Ng and Nicholas, 2013; Song, 2014; Warschauer et al., 2014). There is some disagreement about whether these approaches contribute to inequities (Kobus et al., 2013) with some reports indicating that these concerns are unfounded (Nelson, 2012; Kobus et al., 2013), however tensions surrounding this debate remain.

Teacher stress may also influence the implementation of BYOD in schools (Fogarty and Carr, 2014). Research suggests teachers lack of familiarity with devices (Liu et al., 2014) adds to pressures associated with classroom management and security. With emerging concerns about legal issues associated with ownership of these devices (Sweeney, 2012; Bathon, 2013) it may also be that parents may experience similar management and security challenges in relation to family practices with mobile devices in the home.

## Does BYOD in primary schools enhance students learning?

Although limited, available research indicates that there is merit in the implementation of BYOD approaches and practices in primary schools (see Sweeney, 2012; Johnson et al., 2014). Research undertaken in secondary schools highlights the importance of relationships between parents, students, teachers, IT technicians, principals, and the wider community in contributing to a successful mobile-learning program (Ng and Nicholas, 2013). There are implications for these relationships being also understood in the primary school context.

In a literature review of mobile learning across education contexts K-12 Liu et al. (2014) identified studies where student access to mobile technologies was attributed to blurring boundaries between “formal and informal learning spaces” (p. 357) and extending learning from school into the home. Whether or not this is the case in primary schools is less certain which suggests that more needs to be known about the broader influences of BYOD on family life and school practices and vice versa.

## Conclusion

The implementation of BYOD in primary schools is influenced by local school and family practices, and broader societal trends. This is similar to what Selwyn (2013) describes as the global and local contexts of implementation that has been evident in one laptop per child (OLPC) initiatives. The efficacy and long term sustainability of BYOD in primary schools cannot be determined without first understanding family and school practices in school communities where BYOD approaches are implemented. Future research may inform this process through a focus on understanding experiences from both parent and teacher perspectives. Until then, the implementation of BYOD in primary schools remains open to debate.

## Author contributions

The author confirms being the sole contributor of this work and approved it for publication.

### Conflict of interest statement

The author declares that the research was conducted in the absence of any commercial or financial relationships that could be construed as a potential conflict of interest.
